# Take part in the Grand Challenges in Global Eye Health study

**Published:** 2019-09-10

**Authors:** Jacqui Ramke, Hannah Faal, Matthew Burton

**Affiliations:** 1 Commonwealth Rutherford Fellow: London School of Hygiene & Tropical Medicine, UK.; 2 Chairperson of the Board: Africa Vision Research Institute, Durban, South Africa.; 3 Professor of International Eye Health, London School of Hygiene and Tropical Medicine, UK.

As readers of the *Community Eye Health Journal*, you are warmly invited to take part in the Grand Challenges in Global Eye Health study, which is part of the ongoing Lancet Commission on Global Eye Health, and aims to identify the “grand challenges” in global eye health.

We hope that our results will help to guide future research, and we need your insights and ideas to make this work – whatever your role.

## What is involved?

We want you to answer one question: *What are the grand challenges in global eye health?*

A grand challenge is a “specific barrier that, if removed, would help to solve an important health problem. If successfully implemented, the intervention(s) to address this grand challenge would have a high likelihood of feasibility for scaling up and impact.” Grand challenges may be at a global or national level, or at the level of your clinic or community. Please think about the challenges you see in your daily work and tell us about them.

## What next?

To take part, visit **http://bit.ly/eyechallenge**. Read more about the study in the Participant Information Sheet and scroll down to select whether you do, or do not, consent to take part. The responses you provide will not be presented in a way that could identify you. The deadline is **30 September 2019**.

The study has ethical approval from the London School of Hygiene and Tropical Medicine Research Ethics Committee (Ref 17487). We hope that you will participate in this exciting study and look forward to hearing about your grand challenges. Email: **Jacqueline.Ramke@lshtm.ac.uk** if you would like more information.

